# British South Asian ancestry participants views of pharmacogenomics clinical implementation and research: a thematic analysis

**DOI:** 10.1038/s41397-023-00317-8

**Published:** 2023-11-01

**Authors:** Emma F. Magavern, Faiza Durrani, Mehru Raza, Robin Lerner, Mohammed Riadul Islam, Genes & Health Research Team, Megan Clinch, Mark J. Caulfield

**Affiliations:** 1 William Harvey Research Institute, Queen Mary University of London, London, EC1M 6BQ UK; 2 Genes & Health, Blizard Institute, Queen Mary University of London, London, E1 2AB UK; 3 Focus group participant, London, UK; 4 Wolfson Institute of Population Health, Queen Mary University of London, London, UK

**Keywords:** Health policy, Therapeutics

## Abstract

**Background:**

South Asian ancestry populations are underrepresented in genomic studies and therapeutics trials. British South Asians suffer from multi-morbidity leading to polypharmacy. Our objective was to elucidate British South Asian ancestry community perspectives on pharmacogenomic implementation and sharing pharmacogenomic clinical data for research.

**Methods:**

Four focus groups were conducted (9–12 participants in each). Two groups were mixed gender, while one group was male only and one was female only. Simultaneous interpretation was available to participants in Urdu and Bengali. Focus groups were recorded and abridged transcription and thematic analysis were undertaken.

**Results:**

There were 42 participants, 64% female. 26% were born in the UK or Europe. 52% were born in Bangladesh and 17% in Pakistan. 36% reported university level education.

Implementation of pharmacogenomics was perceived to be beneficial to individuals but pose a risk of overburdening resource limited systems. Pharmacogenomic research was perceived to be beneficial to the community, with concerns about data privacy and misuse. Data sharing was desirable if the researchers did not have a financial stake, and benefits would be shared.

Trust was the key condition for the acceptability of both clinical implementation and research. Trust was linked with medication compliance. Education, outreach, and communication facilitate trust.

**Conclusions (Significance and Impact of the Study):**

Pharmacogenomics implementation with appropriate education and communication has the potential to enhance trust and contribute to increased medication compliance. Trust drives data sharing, which would enable enhanced representation in research. Representation in scientific evidence base could cyclically enhance trust and compliance.

## Introduction

The South Asian ancestry population is a rapidly growing demographic in the United Kingdom (UK), now representing 10% of the national population [1]. South Asian ancestry populations are under-represented in both genomics studies and clinical trials which provide the data that underpin therapeutic licensure by regulators [2–4]. The UK South Asian ancestry population suffers from high rates of multi-morbidity and will therefore be exposed to polypharmacy. This means they are more likely to experience adverse drug events and drug-drug interactions as compared with other populations due to exposure to higher numbers of medications.

Pharmacogenomics (PGx) uses genetic information to predict some of the interindividual variability in response to therapeutics and can help to personalise medication choice to get the right drug to the right patient at the right dose and the right time. PGx can therefore increase efficacy, decrease adverse drug reactions (ADRs), and mitigate drug-drug interactions. The potential benefits of PGx for the UK South Asian ancestry population are substantial, so it is vital engage the community in discussions about PGx clinical implementation and use of generated clinical data for future research.

PGx has potential to address some inequalities by nature of ancestral variation in polymorphism prevalence. For example, it would personalise therapy for those who are poor CYP2C19 metabolizers (higher prevalence in Asian and Oceanic ancestry populations) or ultra-rapid CYP2D6 metabolizers (more likely in those of Oceanic, Ashkenazi Jewish and middle eastern populations) [5, 6]. However, PGx implementation could make inequalities worse if historically under-represented ancestral groups, such as the South Asian ancestry population, do not engage with the PGx research that will flow from clinical implementation [7]. This is because unless there is research engagement from diverse ancestral groups, PGx polymorphisms cannot be validated in diverse populations, and polymorphisms specific to non-European ancestral groups may be missed.

The UK National Health Service (NHS) has committed to examining the evidence for PGx implementation in the next one to three years as part of the national genomic medicine strategy [8].The benefit of patient and public engagement (PPI) in clinical service development is well established. Systematic review shows that care process outcomes emerged from high-level enagagement [9]. Furthermore, engagement can improve the relevance and credibility of research, aligning the research community and research population, and improve accountability to the research population [10].

PPI is critical to shaping and driving PGx implementation. Enhanced research participation from historically underrepresented communities is vital to the goal of using PGx to address health inequality. This is particularly important when there might be disproportionate benefit to historically under-represented communities and potential trust barriers to be overcome.

The objective of this qualitative study is to understand UK South Asian ancestry participants attitudes toward PGx clinical implementation and potential barriers and facilitators in relation to PGx data sharing for research.

## Methods

Due to the lack of any prior PGx public acceptability work in this cohort demographic, focus groups were chosen as a methodology to canvas public input with minimal assumptions.

We recruited to focus groups from existing participants in the Genes & Health cohort study [11]. Genes & Health participants were originally recruited 2015-present in community and healthcare settings [11]. Inclusion criteria were age 16 or older and self-identified as Bangladeshi or Pakistani ancestry. 150 participants who had recently engaged with follow-up studies locally were sent an SMS inviting them to join the focus groups. This was supplemented with invites extended directly to recent or future participants by telephone.

Demographic information was collected from participants in a brief survey administered prior to the discussion. Four focus groups were conducted with 9–12 participants per group. Two groups were mixed gender, one was male only and one was female only. Simultaneous interpretation was available to participants in Urdu and Bengali.

A brief introduction was given on PGx and then PGx clinical implementation and use of clinically generated PGx information for research were discussed.

The focus groups took a semi-structured approach using a topic guide which asked questions about PGx implementation, concerns about taking a PGx test, and sharing clinical PGx data with third parties for research (the topic guide is provided in the appendix). A literature review was undertaken to inform the topic guide development. Though information regarding public and patient perspectives of PGx is scant and high level there are common themes in the literature which served as a starting point for the semi-structured topic guide used (supplementary table 1) [12–22].

A clinician investigator led the focus groups, enabling participants to ask questions about the topic (i.e., how genetic testing samples could be collected). Focus groups were recorded and abridged transcription was performed. The data was analysed thematically, using an inductive approach, to describe perceived utility of and barriers to clinical PGX implementation and subsequent PGx research [23].

This study was approved by the Queen Mary University of London Research Ethics Committee (QMERC22.353). Written informed consent was obtained for participation in the study. Participants were given a 50 GBP voucher to thank them for their time and participation. A member checking session was held to discuss the results of the thematic analysis.

## Results

### Focus group demographics

There were 42 participants across the four groups, 64% female. 26% were born in the UK or Europe. 52% were born in Bangladesh and 17% in Pakistan. 36% reported university level education. More detailed information to characterise each focus group is shown in Table 1.

**Table 1:** Detailed demographic information for focus group participants.

Focus Group	1	2	3	4
Number of participants	12	12	10	8
Female Gender (%)	70% (9)	100% (12)	60% (6)	0% (0)
Average age (Range)	37 years (18–45)	42 years (23–59)	33 years (21–43)	35 years (16–48)
Spoken Language				
English	67% (8)	42% (5)	50% (5)	50% (4)
Bengali	17% (2)	58% (7)	50% (5)	13% (1)
Urdu	17% (2)	0% (0)	0% (0)	38% (3)
Born in				
UK	25% (3)	8% (1)	50% (5)	0% (0)
Bangladesh	25% (3)	83% (10)	50% (5)	50% (4)
Pakistan	33% (3)	0% (0)	0% (0)	38% (3)
India	8% (1)	0% (0)	0% (0)	0% (0)
Other	0	8% (1)	0% (0)	0% (0)
University education	75% (9)	25% (3)	30% (3)	25% (2)
Country of education				
UK	25% (3)	0% (0)	50% (5)	25% (2)
Bangladesh	25% (3)	25% (3)	10% (1)	25% (2)
Pakistan	33% (4)	0% (0)	0% (0)	0% (0)
India	8% (1)	0% (0)	0% (0)	0% (0)
Other	0% (0)	8% (1)	0% (0)	0% (0)

Some participants did not respond to some questions; therefore, percentages do not always add up to 100%.

### Thematic analysis

Main themes that emerged are shown in Table 2. For PGx clinical implementation these were: benefits, communication, timing of testing in the clinical care pathway, custodianship of data, cost, trust, education and outreach. Themes that emerged from discussion of sharing clinical PGx data for research purposes were: benefits, trust, education, data sharing facilitators, barriers to data sharing and safeguards. Themes were consistent across all groups, and all groups emphasised trust as primary and interlaced with all other themes.

**Table 2:** Themes and sub-themes from inductive analysis of the four focus groups.

	Themes	Sub-themes
Clinical implementation (1.0)	Benefits (1.1)	*Which medicine ‘suits’ me (1.1)* Reduced side effects, higher efficacy, more personalised to each individual and diverse communities
	Communication (1.2)	*Communication to support clinical PGx implementation (1.2)* Simplicity, Person communicating, differentiate from diagnostic testing /disease prediction
	Timing (1.3)	*Timing of testing in the clinical pathway: Who would benefit the most and how should testing eligibility reflect that? (1.3)* When to offer, at what stage of illness/health
		*Testing in primary care. (1.31)* Where in care setting/journey
	Custodian of data (1.4)	*Maximising benefits of clinical PGx testing: transfer of information across care settings. (1.4)* Who keeps test results, how do they travel
	Cost (1.5)	*Balancing benefits against costs (1.5)* Direct costs, indirect costs
	Trust (1.6)	*The role of trust in clinical PGx implementation: ‘GP they trust’ (1.6)* Factors contributing to lack of trust, how to build trust
	Education and Outreach (1.7)	*Education to support clinical PGx implementation (1.7)* Educational needs and baseline awareness
		*Outreach and engagement (1.71)* where to outreach, use local community members to lead engagement
		*Education and misinformation – lessons learnt from the covid-19 pandemic (1.72)* Emerging evidence and shifting practice through the lens of the covid-19 pandemic
Research (2.0)	Benefits (2.1)	*Benefits of research using PGx clinical data: ‘whatever is necessary to help the community’ (2.1)* Improved medicines use in future, for this community specifically
	Trust (2.2)	*Trust in PGx research: protective and harmful factors (2.21)* Concerns around sharing data with different groups or institutions
		*Lack of trust leads to concerns about data misuse (2.22)*
		*Lack of trust in profit driven research (2.23)*
		*Feeding back research results facilitates trust (2.24)*
		*Trust in therapeutics through the lens of the covid-19 pandemic (2.25)*
	Education (2.3)	*Education to facilitate PGx research (2.3)* Lack of understanding of genes/DNA
	Data sharing facilitators (2.4)	*Factors supporting PGx data sharing for research. (2.4)* Trust, lack of conflict of interest, benefit sharing
	Barriers to Data sharing and Safeguards (2.5)	*Barriers to sharing clinical PGx data for research and potential safeguards (2.5)* Concerns about privacy, data ownership and data misuse for profit. Gating of information, protective legislation and grouping of potential access groups were suggested safeguards.

**Fig. 1:**
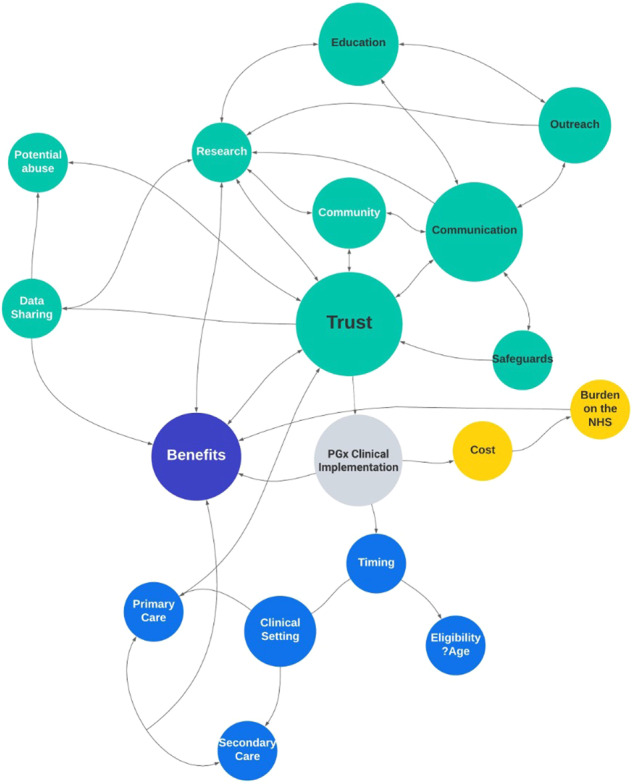
Mind Map of focus group theme and sub-theme interactions. PGx Pharmacogenomics.

The relationships between these themes are illustrated in Fig. 1 (Fig. 1- mind map).

These key themes are expanded on below, with sub-themes, to illustrate participant insights (Table 2).

#### PGx clinical implementation

##### Benefits

***Benefits of clinical PGx implementation: which medicine ‘suits’ me***

PGx was perceived to be beneficial to individuals, by making medication choice more tailored, with less trial and error: “*which medicine suits me, I think that would be a good idea*”. There was particular interest in implementing PGx for gene-drug pairs where there are known to be high prevalence of polymorphisms in South Asian ancestry groups, and therefore a higher risk of inefficacy or toxicity in this community. Risk of ADRs were perceived to be a big concern in taking medications, and to impact on compliance. There were concerns that ADRs can be worse or more long-lasting that the original treatment indication, and that if participants knew of someone who reacted badly to a medication, they would not want to take it:

*“For example, I take a medicine and I react really badly to it. Everyone in this room might sit there and think, wow, she’s taking that medication and she’s had a really bad reaction. Maybe I shouldn’t take that medicine.”*

Participants felt that PGx had the potential to mitigate this reaction by reassuring people that genetic risk of ADRs had been checked.

The potential to avoid broad contraindications with a more targeted approach was raised by several participant anecdotes. For example, one participant suggested that with more precise PGx stratification we might better understand which asthmatics are likely to have a bad reaction to ibuprofen and not withhold it from those who are not likely to have an ADR.

##### Communication

***Communication to support clinical PGx implementation***

Participants felt that limited information was desired for clinical PGx use at the point of care. There was a strong preference for use of simple language to communicate PGx. Participants thought that the easiest way of conveying the utility of PGx was to identify which medicines “*suit*” you/your body. Participants generally agreed that for clinical indication a minimal explanation of PGx testing to inform medication choice (similar to a routine blood test) was sufficient. Many participants didn’t think it was necessary or helpful to include the fact that DNA/genetics are being tested. For example, as one participant reflected elderly people might not understand what genes are in comparison to younger people. Given this, they suggested presenting PGx as something that would help clinicians make sure that the medicines they prescribe are “*more suitable for you*” would result in an explanation that would make sense to a wider range of people. This sentiment of offering PGx clinically for medicine optimisation without detailed discussion of genetics was echoed by the majority of participants across all focus groups.

There was a strong preference that communication around PGx be led by General practitioners (GPs). GPs were described as trusted sources of information and having the skill and resources to support communication where language and literacy barriers are present: “*GP can explain very well”*.

##### Timing

***Timing of testing in the clinical pathway: who would benefit the most and how should testing eligibility reflect that?***

PGx was viewed as particularly helpful to those who suffer from polypharmacy. People taking many medications were perceived as most likely to benefit from PGx testing, by decreasing risk of side effects and drug-drug interactions. In addition to identifying polypharmacy as increasing risk of ADRs, participants felt that enhancing efficacy from medication for those with the most morbidity was important, regardless of age. In the words of one participant, which provoked broad agreement “*you could have someone that is like half the age and has already been using so many different medicines. They aren’t working for them and they wanna know why it’s not working*.”

Due to the shared view amongst participants that the greatest beneficiaries of PGx implementation would be those with the most morbidity, they proposed the idea of a secondary prevention speciality clinic. They felt that this would mean that people at high risk would be able to benefit from PGx innovations “*as soon as possible*” rather than having to wait potentially many years for pre-emptive PGx to be rolled out across everyday clinical practice for all people via NHS primary care.

While benefits of more personalised medicine were thought to be particularly promising for multimorbid patients, if resources allowed participants liked the idea of PGx panel testing for all at birth, so that the information would be there pre-emptively to optimise medication choice throughout life. Several participants suggested they would welcome PGx testing as a part of routine neonatal testing: “*you know the kids are born and then they offer the next day the hearing test …in the hospital without leaving? You can offer [PGx] at the same time*.” Participants had no concerns about doing PGx testing on babies, provided sample extraction was not painful or harmful. Parents were much more concerned with the risks of a perceived trial and error prescribing approach that did not consider genetic data which could indicate high ADR risk.

***Testing in primary care*** *.*

Participants felt strongly that pre-emptive PGx testing via the GP was preferable to point of care testing in hospital at the time of indication for therapeutic (ie in the example of *CYP2C19* testing to help guide anti-platelet choice after a myocardial infarction). The reasons were multifactorial; the GP was first point of contact, had all patient information, provided continuity of care, and was perceived to communicate well. Participants liked the idea of having PGx testing before there was a treatment indication, and felt primary care was the right place for this kind of anticipatory testing. There was also a concern that anything viewed as not essential may not reliably happen in acute care settings. Furthermore, participants thought of primary care as a less threatening and more personal setting where there was a higher likelihood of receiving information about test results and being in a state to understand that information, as compared with hospitals. “*Going to the GP… it’s a lot more personal than going to a hospital… if you’re at a hospital it just kind of feels alien*”. They also felt that need for acute care was associated with fear: “*people go in hospital when [they are] in danger…I can call the GP and book an appointment… when you go to hospital [there’s] always danger there*”. Participants felt that due to the acute nature of secondary care communication was limited, and patients were often unaware of investigations ordered. As one participant surmised: “*We don’t even know probably half of the things they do. No one questions about the medicine or why they’re taking the blood test. There’s no choice*”.

##### Custodian of data

***Maximising benefits of clinical PGx testing: transfer of information across care settings***

Benefits of PGx were thought to be greatest if PGx results could be effectively shared across care settings, particularly primary and secondary care but also community pharmacy settings. Some participants felt that integration across care settings of existing analogous data is not good. One parent illustrated this with an anecdote:

*“I have an example: One of my sons [is] allergic [to] ibuprofen. So, this information I can see …the GP shared with me…but always I have to tell [them] in a hospital, don’t give ibuprofen to him, because he has a reaction with that”*.

However, another participant gave examples of successful programmes where important medical information travels with patients, suggesting the same could be done with PGx: “*Shouldn’t be a problem because you already have medical bracelets and tags for people with different…conditions… so they could be identified if some something was to happen to them in public you can see that necklace or bracelet*.”

Participants liked the idea of an NHS app having PGx information that could travel with the patient and allow self-advocacy. For example, in the words of one participant:

*“It should be the GP as well as the patient who has that information because… sometimes… the GP don’t really listen properly… if she knows what her needs are… she can show it and say this is what it is. This is my genetic result”* (translated from Bengali).

Some participants saw community pharmacists as care providers that could give more personalised advice if they had access to PGx results. This could take some strain off primary care. However, others perceived sharing clinical PGx data with private chemists as a risk that could lead to inflated prices.

##### Cost

***Balancing benefits against costs***

The benefits of clinical PGx implementation, particularly as pre-emptive testing for all nationally, were weighed against the risk of overburdening resource limited NHS services and clinicians, which participants felt protective of. There was trust in the NHS and NHS clinicians and a perception that the benefit of PGx implementation would need to outweigh added financial strain and time constraints on these institutions and professionals.

There was a feeling that any preventive endeavour would be lower priority, as compared with testing which responded to clinical need. In the words of one participant, which the other participants expressed agreement with:

*“you know they’re suggesting a GP visit should cost people money…what about the cost of the test… would it cause too much pressure? …In advance you are doing a testing… Maybe you need it in the future or not…still you are doing it … they’re asking for less pressure on the NHS then you’re putting so much more pressure on the NHS.”*

Many participants felt that streamlined logistics of PGx implementation were crucial to ensure the inconvenience of participation wasn’t perceived to outweigh the benefits.

A further concern to the integrity of existing services and professionals was any added threat of litigation. This concern further highlighted the protective feeling participants had toward the NHS, and the requirement that the benefits outweigh the all-inclusive costs:

*“Could this open up the NHS or the GP to liable action, i.e., being sued? Because they have the genetic markers there. You gave the medicine, but now obviously they got it wrong…Patient then sues the GP/brings action against the NHS because you’ve given me a wrong medicine, even though you’ve had my genetic markers.”*

##### Trust

Trust was a central theme in discussion of clinical PGx implementation and was impacted by and impacted on communication and education.

***The role of trust in clinical PGx implementation: ‘GP they trust’***

There were strong feelings of trust in the NHS and health care providers, particularly primary care practitioners. Examples of broadly shared articulated trust in GP were common. Despite this trust, participants commonly cited concerns about side effects leading to medication non-compliance. “*Some people are quite scared of taking any medication because of all the side effects. Even if they get the medication from the GP… they’re going to ask how many side effects [and then] don’t take it”*.

Participants thought that a more personalised approach to prescribing using genetic information would enhance trust in prescribers and prescriptions, because people would have more confidence in the selection of therapeutics knowing it was aligned with their personal test results. “*After genetic test when doctor will prescribe medicine obviously there’re going to involve more trust on this*”. Participants thought that this enhanced trust would improve medication compliance, as demonstrated by one participant:

*“For example, if I go doctor then they just prescribe me paracetamol? Yeah. If they tell me. OK have 100 [dose]. Maybe I’m gonna have 20 or 30. But after the blood test or whatever test done. If he give me 100 then I’m gonna say yeah I’m gonna finish the 100 because it’s been done by test… In the first time, he gave me 100, I’m not gonna take it.”*

Ancestry specific representation in research generated evidence for therapeutics was noted to build trust in a clinical setting: “*If you get a medication out and say we tested it on these kind of… people… and that was beneficial… This drug was good for Asian community… So it’s better to take that.”* The implication was that participants know that ancestry is sometimes linked with response to medication. Therefore, proportionate ancestry representation in evidence base assessing efficacy and ADRs builds trust in clinical practice by demonstrating that a specific medicine has a favourable risk-benefit profile in their community. Due to trans-ancestry variation in pharmacogene polymorphisms and historically non-diverse clinical trial cohorts this is an important point in how clinical PGx implementation interfaces with trust.

Interestingly, there were no concerns from participants around misuse of data within clinical care pathways. Participants unanimously felt that their clinical data was secure through standard NHS data protection pathways and that PGx data would be no different. In the words of two participants: “*I think the GDPR legislation makes me comfortable with sharing my information with the GP and the NHS, so I don’t see any hindrance…sharing my information*”; “ *the current GDPR is quite broad*”. However, participants felt that any sharing of personal data with private entities such as chemists could result in price gouging if, for example, pharmacies discovered they were serving a population who were much more likely to respond well to one specific medication. This was a widely shared concern.

##### Education and outreach

***Education to support clinical PGx implementation***

There was consensus that national roll out of pharmacogenomic testing should be accompanied by public health level education, with outreach, and clear communication to facilitate trust. It was clear from the focus group discussions that it is important to differentiate diagnostic genetic testing or genetic testing to predict disease risk from PGx testing. There was a general concern from participants that the level of genetic literacy in the UK South Asian community is low. There was a feeling that people with more lived experience of disease and medication use were more likely to understand and be interested in PGx.

***Outreach and engagement***

Participants universally acknowledged that GPs would not be able to discuss PGx with each person. This was an impetus for support for a broader public health and outreach awareness campaign proposed by participants.

Forums such as local mosques, Islamic centres, schools, fairs, shopping centres and GP surgeries were suggested to disseminate information about PGx. “*The mosque …some Islamic mosques have community services [centres] as well… the kids there are learning…the elders are coming there…women are coming there…mosques have a community system…the ladies are very much involved in that*.”

Multi language leaflets and videos were enthusiastically suggested, as was propagation of information via social media. The importance of leadership in the community, and community and family links, were paramount. Therefore, secular and faith leaders and heads of family were perceived to play a key role in propagating information. There was also a suggestion from participants in every group that information can be disseminated in families by incorporating education about genetics generally and PGx specifically in schools. “*Getting children to understand…maybe they can go home to their parents…if you come to schools and talk about it*”.

***Education and misinformation – lessons learnt from the COVID-19 pandemic***

Participants framed their experience with dissemination of new medical information through experiences with COVID-19 vaccines. There was a broadly shared view that misinformation around the COVID-19 pandemic and vaccines had eroded trust between the community and health care. There was perceived to be a new reluctance to engage in any non-essential clinical tests:

*“You know covid changed everything. Do you think that people will go for the blood test or genetic test that don’t know why you are using this, why we need this? So you need to educate them what is the importance for them. Otherwise, it’s very hard for the Asian community to come.”*

The pandemic highlighted a need for high quality accessible information regarding new developments in therapeutics related clinical care (“*to spread information and minimise misinformation*” in the words of one participant), and ability to understand which demographics different forms of information was reaching. Misinformation was a concern, particularly via social media platforms, where it can be widely disseminated: “*There’s so much data on the internet, and so much information it can be false*”. Education with outreach was seen as a solution to the problem of misinformation. Social media was seen as an effective tool to combat misinformation and democratise knowledge via accessible multi-media campaigns.

#### Sharing clinical PGx data for research purposes

Themes that emerged from discussion of sharing clinical PGx data for research purposes were: benefits, trust, education, data sharing facilitators, barriers to data sharing and safeguards.

##### Benefits

***Benefits of research using PGx clinical data: ‘whatever is necessary to help the community’***.

Research that could be generated from use of PGx clinical data was felt to be beneficial to the community with some risks to the individual’s privacy. Participants felt favourably about contributing data to support research which would benefit the community, and the good of the community had a central role in discussion. As one participant said, and others echoed: “*What’s the point in just having the blood test done and not going for research. I think that goes hand in hand…I would take it… Whatever is necessary to help the community*”. However, there were strong feelings about privacy and concerns that any data sharing may breach privacy and open potential for misuse:

*“I think data protection is very important in our lives…Yeah like how we said it should be between …researchers and GP…I don’t think I would like everyone to know… what benefits me… I would like to have privacy ourselves as well”*.

These privacy concerns were counterbalanced by the benefits of community representation in research to develop community specific knowledge. A participant highlighted concern that research on medicine is only done in some people, but then the medicine is used in all people, and participants agreed broadly that it is reassuring to be treated with medicine when the evidence base for medicine use included their community. “*When scientists do research there is one portion of the population but how [do] they apply that information onto the big portion of the population?*” (translated from Urdu).

Participants felt more favourably about taking medication that had been trialled in their ancestry group and felt that ancestry specific research could drive changes in medication or supplement taking behaviour. For example, a participant gave an example of impact on behaviour driven by community specific research: most people in the community didn’t take vitamin D supplements, and then research that South Asians often lack vitamin D was disseminated. This research specific to the South Asian community then convinced many people in the community to take vitamin D: “*They got some information Asian people lack vitamin D. Apparently it’s in the genes or something…majority of the Asian people, my family members, all of them, they take vitamin D”*.

Benefits of data sharing to generate further research specific to the South Asian community was perceived as outweighing the potential risks of data misuse generally, particularly with appropriate safeguards: “*if it benefits the community by sharing the data… with their permission, with their consent, if this is shared in the research team that’s fine also… keeping data secure, confidential with her permission*.”

##### Trust

***Trust in PGx research: protective and harmful factors***

Willingness to share clinical data for research purposes revolved around trust. Participants felt that more personalised therapy through PGx clinical implementation would enhance trust and therefore contribute to increased willingness to engage with and share data for research. Trust was engendered by institutional affiliations (i.e., NHS, medical practitioners, national regulatory bodies such as the Medicines Healthcare Regulatory Association (MHRA)). Trust was supported by safeguards in data protection and de-identification of data used for research. Participants also found the non-diagnostic nature of PGx testing reassuring, and keeping the scope of PGx testing to non-disease diagnostic genes was a factor that enhanced trust: “*I feel if like it’s really narrowed down in front of you it would be safer …”*. Trust in the individual recruiting to research was also a factor. Trust leading to research engagement could be gained by endorsement of a family member, faith leader, or community leader: “*If my relative did it, I might [do it]. Some people trust in relatives…People trust more family*”.

Trust was harmed by insecure data, a history of data breach or association with individuals or institutions that were not trusted. Lack of consistency in information and profit as a motivating factor were other factors which harmed trust.

***Lack of trust leads to concerns about data misuse***

Misuse of data by non-trusted entities was a concern. This was a central disincentive to research participation: “*People really don’t want to share their information. They might have doubt on the people using to do research. That’s why they don’t want to share*” (Translated from Urdu). Concerns regarding the specific nature of potential data misuse ranged from breaches of privacy and financial exploitation to the potential for malicious actors to use genomics data for racially motivated genocide.

*“In theory… if someone wants to target a …specific group of people like South Asians… if I target that gene it could set off a virus that could only affect these people…I think I’ve seen it in a film, when they target a specific gene … they set this gas off but it will only effect people with this gene…South Asian genes”*.

This latter was perceived by some participants to be hyperbolic, and the level of time, knowledge and resources needed to misuse data in such a nefarious way were cited as protective: “*to get to the point of …killing hundreds of thousands… is far-fetched. We’d need to dissect …an entire genome, which would take a very long time, and a lot of work*.”

***Lack of trust in profit driven research***

Participants across all groups expressed concern that pharmaceutical industry was not trustworthy due to profit as a motivating factor. “*Medicine is about helping people and saving lives…They’ve developed the drugs but they’re big businesses as well*…”. Some extended this logic to private chemists and pharmacists working at chemists, as profit was felt to be the bottom line. The perceived conflict of interest created by profit as a primary goal was felt to lead to risk of information misuse.

There were concerns about benefit hoarding for profit. Many participants across all groups worried that if industry were to get PGx data for research they would find a way to profit at the expense of the community and withhold benefits from the community. Several participants felt there was a risk of price gouging if a therapeutic was found to be particularly beneficial to their community:

*“but when the makers know that then they will increase the prices. And you know we are very careful about our health so we will spend money.”* Another participant in a different group expressed the same sentiment: *“If this information is being delivered to industry, will it affect the cost of the medicine?… if we’re getting a tablet for 1 pound we might then get it for 3 pounds”* (translated from Urdu).

There was a negative view toward proprietary patents as tools to restrict availability of therapeutics. There was a concern that if lifesaving medications were discovered from genetic data, patents would mean that the medicine would not be affordable or accessible to the participant communities that had contributed data to the research.

However, trust in national regulators was seen by some participants to counterbalance the risk of unrestrained industry: “*business is business at the end of the day. Some businessmen are OK. If the regulator doesn’t allow, then they won’t get it [the medication]. They need to allow it first*.”

***Feeding back research results facilitates trust***

Feeding back research results was crucial to ongoing research engagement through building trust: “*if someone sees a result then they will become more involved*”. Participants agreed that if research results were fed back it would support education and engagement and facilitate trust via grassroots community communication. As one participant said of receiving feedback on how she contributed to a study: “*And then you would speak to, like, your friends and family…They would open up. They would be like, wow, that’s so cool… It would build trust between communities*.” Some participants felt that personalised feedback on an individual level had an even more powerful impact, and there were suggestions that researchers could build trust further by contacting individual participants to make them aware of how their data had contributed to a study.

***Trust in therapeutics research through the lens of the COVID-19 pandemic***

Participants expressed their experience with trust, and mistrust, in therapeutics and research through the lens of the COVID-19 pandemic. Participants reported a change in context and trust toward therapeutics research due to the pandemic. There was broad agreement that lack of trust had manifested in strongly divided opinions on the safety and efficacy of the COVID-19 vaccine:

*“for example…, covid injection, half of the people …didn’t have it…a lot of people I know, they didn’t go for that injection…it’s their choice end of the day…but there’s a reason why they didn’t have it…because they don’t trust maybe, they didn’t believe”*.

Lack of trust toward the COVID-19 vaccine within the South Asian community was widely felt to be prompted by the perceived pace of research and social media reports of trusted health care practitioners refusing COVID-19 vaccination. Because of the nature of the pandemic, some participants saw COVID-19 vaccines and treatments as initially experimental or offered without a full understanding of possible effects. However, it was felt that this mistrust would not extend to PGx research focused on optimising personal risk/benefit profile for existing medications.

##### Education

***Education to facilitate PGx research***

In contrast to the skeletal information desired for clinical PGx use at the point of care, participants felt that a lot of information and education was needed to responsibly organise sharing of the generated clinical data for research. “*for research: you have to make sure you understand it perfectly and it has to be accurate information given to you. Clinical, that’s something you just do…easier to do…research you have to be really accurate*.”

Participants highlighted lack of awareness of research, and lack of scientific and genetic literacy as significant barriers to research engagement. *“this is the reason there is less data from these groups: because the lack of education and they don’t participate if they don’t understand anything.”* Language barriers were also cited as key hurdles to engagement of this community with research.

However, participants also perceived a lot of interest in advancing health and medication related knowledge in the community and suggested that community ties offered vehicles to public engagement. The public health engagement campaign suggestions outlined above around national PGx roll out were echoed strongly in the discussion of education to facilitate PGx data sharing for research. Engaging with local community members for grass-roots education was advised by participants. But some participants perceived a lack of scientific literacy to be a significant barrier to community exchange of information:

*“How to educate those people? Like when you speak to other people they don’t know, like when she will leave from here, what she would say to her neighbour … what is that genetic information to do with the medication? We take medication everyday”* (translated from Urdu).

Many participants expressed interest in being trained to be community champions and volunteered to disseminate PGx information to facilitate research engagement: “*in East London mosque they have events and things… I’m here today. I understand. I will go and spread to my friends and family. So, it’s like word of mouth will get spread*.”

##### Data sharing facilitators

Data sharing was the key concept on which research from a hypothetical clinical PGx service hinged. Participants required prompting to distinguishing PGx testing for clinical use from sharing clinical PGx data for research.

***Factors supporting PGx data sharing for research***

Data sharing was desirable if the researchers did not have a financial stake, and benefits would be shared. There was a common perception that without research, the use of medicines will not improve, but an understanding that the risk is to the participating individuals while the benefit would be for future individuals:

*“If you don’t share it, you don’t advance really. So, you have to come to some sort of compromise where you are sharing the results they need, or do you want to just not share it and be stuck and not give two hoots about what happens in the future. You have to draw that line somewhere.”*.

The perceived “good” of the research purpose was a key motif: “*So the point is how it works when we share for the good purpose, not for the bad purpose. So, it can help, so definitely [we should] share*”. There was broad consensus across individuals and groups that the idea of good as compared with bad purpose had an association with the trustworthiness of the researchers: “*we have a concern, so we can only share these things [PGx data] with trusty [trustworthy] ones and [make ourselves aware*].” The perceived trustworthiness of both individual researchers and associated institutions were determining factors in weighing willingness to share clinical PGx data for research purposes.

Health care professionals, academic institutions, and the regulatory body (the MHRA) were considered trustworthy and therefore participants were happy to share PGx data with these groups for research with the protection of standard data de-identification and data protection.

##### Barriers to data sharing and safeguards

***Barriers to sharing clinical PGx data for research and potential safeguards***

Participants across all groups broadly acknowledged that some people would not like to share data as a rule, due to privacy concerns: *“There are people with those [privacy] concerns and those concerns are very real”*. Data ownership was an important topic linked with privacy, and many participants wanted to maintain control over access to their data *“It’s my information. That’s mine, do you know what I mean, it’s an invasion of privacy where you don’t have control over who gets to see your information.”*

As compared with healthcare practitioners and academics, there were very different perspectives on sharing PGx data for research with industry. Concerns revolved around trust, as outlined above. Most participants felt that industry has an inherent conflict of interest as a profit driven private enterprise and therefore could not be trusted to prioritise benefit sharing/the health of the community over potentially exploitive options: “*pharmaceutical companies are only thinking about their profits then it’s not good to share our information with them*”.

Others felt that there is an inherent risk to not doing research: “*without research there will be always risk, there’s no cure*”. Some perceived the benefits of data sharing with industry to outweigh potential harms: *“It improves the medicine, so it improves the patient care*”.

Confidentiality and anonymization of data were important safeguards to protect privacy. “*It’s anonymous isn’t it…so the people who are doing it, they don’t know who it is. They have to have that barrier that [data] is confidential and not to be leaked to anyone*.” Well-articulated policies around data protection and management of any breach of data protection were perceived by many participants to be crucial safeguards: *“It’s important what they’re going to do with the data but also if there is a breach of data, what happens… if they find that information was leaked to the public…what they do*”.

Transparency about potential conflicts of interest and opportunities to opt out of data sharing with non-trusted research partners were desirable.

*“I think everyone should be given the option to opt in and opt out, so I think that’s potentially a way of going forward … so you [can] opt in for pharmaceuticals or universities and … and so on…You can label them as non-profit organisation and for-profit organisations and so. That would build confidence in the person that is being involved in the research.”*

Safeguards against financial exploitation due to knowledge of individual or community PGx data would be protective.

## Discussion

There were key cross-cutting themes common to discussion of both clinical implementation and use of clinical data for research. These included: benefits, the central role of trust, concerns about baseline education and desire for public heath level campaign to address this perceived need, and data sharing/custodianship. These echoed existing themes in the literature around the central importance of public awareness, education, trust, and data custodianship (Supplementary table 1). However, the interaction between the key themes across clinical application and research domains was rich, particularly around trust, and adds some novel detailed insight around building trust within this population.

**Fig. 2:**
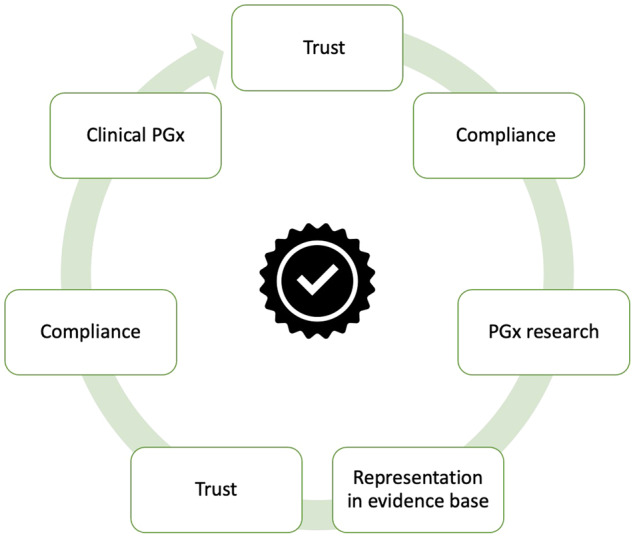
A circular trust building and benefit model of pharmacogenomics clinical implementation and research. PGx Pharmacogenomics.

PGx implementation with appropriate population wide education and clinician communication was perceived to have the potential to enhance trust in clinical care systems by personalising therapy to individuals, particularly those from under-represented ancestorial groups. This increased trust was thought likely to contribute to increased medication compliance. Trust drives willingness to share data and engage with research, and participants linked increased trust in clinical prescribing with increased willingness to share data toward advancing PGx because they could see PGx benefits in action (i.e., there is clinical value proven from PGx research). Representation of the South Asian ancestry group in therapeutic evidence base through research increases trust in the evidence base for medicine use and may increase compliance with therapeutics. Therefore, participants constructed a circular trust building and benefit model that could see a well implemented PGx roll out promote increased medication compliance via trust in clinical systems and increased research representation, which would then feed information back into clinical practice, further supporting trust (Fig. 2). The relationship between participants and GPs were key to promoting this model of trust, as was feeding back utility of research to those who choose to participate, public health level education campaigns, and stakeholder guided data gating.

Therefore, if the NHS decides to adopt panel PGx testing nationwide, educational and engagement initiatives should proceed the roll out, with accessible materials in multiple languages that can be disseminated either by championing individuals or via multi-media/social media. Engaging with community leaders to disseminate information is a valuable approach, as well as optimising intergenerational information sharing by educating those in school.

Success of a national PGx programme is likely to hinge on the level of trust built into the rollout. Some of that trust is engendered already by trusted individuals, professionals, and associations, but some must be earned by education and engagement initiatives with the public. The COVID-19 pandemic demonstrated how easily misinformation can be disseminated and erode trust. The unanimous emphasise on mistrust kindled by the COVID-19 pandemic have implications for PGx, particularly in Black, Asian and minority ethnic groups, not prior discussed to our knowledge. These findings highlight the importance of building from existing trusted relationships with GPs and carefully considering stakeholder suggested safeguards to preserve trust.

Trust can be supported by robust and transparent policies around protection of data, management of data security breaches, and stakeholder input on proposed data sharing. Sharing any data which could be used by private entities for fiscal gain is likely to be a particular source of contention and therefore should be continually informed by stakeholder consultation. Policies that would protect against price gouging as a result of proprietary gains from clinical PGx data sharing for research should be considered.

This study suggests that pre-emptive PGx roll out via primary care is the preferred approach in the long term, but participants highlighted secondary care prevention clinics as a high benefit population in which to pilot panel PGx testing.

### Strengths and limitations of this study

This is the first study to engage UK participants of South Asian ancestry in discussion of facilitators and barriers to PGx implementation and secondary research. Further research should be done quantitatively to canvas large scale public awareness and attitudes to PGx clinical implementation, utility, and sharing PGx data for research in this community. The study was made possible by collaboration with the Genes & Health research team and their links and pre-existing trust building with the community. However, participants recruited from a cohort who have chosen to participate in a large-scale genetic research study may not be representative in their attitudes toward PGx.

### Clinical implications

This participant data from an under-characterised and disproportionately morbid population within the UK is valuable to influence policy on PGx implementation. Inclusive engagement studies can increase the likelihood that PGx implementation would become a tool to improve the health of this group at high risk of polypharmacy and support underpinnings for data sharing to generate PGx research specific to this under-represented population. Such a stakeholder informed approach will support PGx to be a tool which reduces instead of exacerbating health inequality.

### Supplementary information

Supplemental Material

## Data Availability

As specified by our ethics approval, the unanalysed data will not be available to ensure the confidentiality of our participants.
