# Supplementary material for: British South Asian ancestry participants views of pharmacogenomics clinical implementation and research: a thematic analysis
Source: Pharmacogenomics J. 2023 Nov 1;23(6):185–94. doi: 10.1038/s41397-023-00317-8 (PMC10661738; doi:10.1038/s41397-023-00317-8)
Supplement: Supplementary file 1 — Supplemental Material [file 41397_2023_317_MOESM1_ESM.docx]

Supplementary materials

**Supplementary Table 1:** A review of common themes from studies investigating pharmacogenomic implementation from the perspectives of patient/public, prescriber, or a mixed group^1–11^.

| **Study population** | **Patients/Public** | **Prescribers** | **Mixed** |
| --- | --- | --- | --- |
| **Common themes** | Holistic approach to diagnosis and medication use  Concern re prevalence of ADRs at present – PGx could help  Cost effectiveness was a concern  Storage and privacy of genetic information was a concern  Patients would want a high level of information and valued effective communication  Participant driven counseling needed  Trust was important, trust in research, trust in doctors, trust in pharmacists  Experience with healthcare and health was important to inform significance/relevance  Perceived potential harms ie less effective or more expensive medication, insurance implications  Want to receive information specifically tailored to their health vs general PGx info | Education of primary care workforce  Ethical, legal and social aspects -impact on patients  Health economics  Informatics  Testing timeframe  Patient acceptability  Adherence perceived as much bigger problem than PGx  Sensitivity to all thing genetics related in tribal settings | Education for public and clinicians  Lack of evidence for clinical utility  Reimbursement  Data registration and sharing  Decision support tools  Responsibilities ie doctor vs pharmacist  Cost effectiveness  Infrastructure to support testing and interpretation  Turnaround time of testing  Effect on family members |

**Appendix**:

Topic guide

**Session introduction and overview – introductory information given about PGx, and the example of CYP2C19 testing for Clopidogrel was discussed.**

1. Do you think it is a good idea to test people to see if their *CYP2C19* gene works properly? If so, why?

2. If you think it is a good idea, when would you prefer to be told this information:

-By you GP during a routine appointment in which your heart attack risk was explored?

-After a heart attack when you are being treated in hospital?

-What is your reason for choosing one of these options over the other?

3. If you knew this genetic information about yourself, what would you like to be able to do with it?

-For example, would you like to be able to share it with healthcare providers or pharmacists?

4. If you would like to be able to share it with people, how would you like to do this? For example, stored on an app on your phone?

5. Would you like information like this to be stored on your electronic healthcare records so healthcare professionals who prescribe medication were aware of it? Doctors in hospital? GPs? Pharmacists? Please explain your answers to this question.

6. Do you have any worries about using genetic information to inform the medicines you and others are prescribed? Please explain any concerns you have or why you are not worried about this. What factors would make you decline a PGx test?

7. Do you have any suggestions for how healthcare professionals can explain PGx results to patients well?

8. If you had a side effect to a medication, would you be willing to report your symptoms to the NHS?

-If yes/no, why?

9. If you were willing to record your medicine responses, how would you prefer to do this? For example, by speaking to someone such as your GP, a person who works in a hospital? Or by a phone app or an online form?

-What is the reason for your answer?

**Expanding the Evidence**

10. Can you think of any reasons why people would not want to be involved in PGx research?

11. How might we work with people to encourage them to become involved in PGx research? Are there any concerns that may be particular to your community?

12. Would you be willing to have genetic information related to medication effects shared with academic researchers so they could design or improve medications safety and effectiveness?

-Would you be willing for your genetic information to be shared with the medication regulators in the UK when they look at reports of people who have suffered harms from medications?

If yes/no, why?

-Would you be willing for your genetic information to be shared with pharmaceutical companies when they are developing treatments.

If yes/no, why?

References:

1 Meagher KM, Curtis SH, Borucki S, Beck A, Srinivasan T, Cheema A *et al.* Communicating unexpected pharmacogenomic results to biobank contributors: A focus group study. *Patient Educ Couns* 2021; **104**: 242–249.

2 Andrea Smith and Hannah Loshak. *Pharmacogenomic Testing for Medication Selection: A Rapid Qualitative Review*. Canadian Agency for Drugs and Technologies in Health, 2020.

3 Qureshi S, Latif A, Condon L, Akyea RK, Kai J, Qureshi N. Understanding the barriers and enablers of pharmacogenomic testing in primary care: a qualitative systematic review with meta-aggregation synthesis. *Pharmacogenomics* 2022; **23**: 135–154.

4 Rigter T, Jansen ME, Groot JM de, Janssen SWJ, Rodenburg W, Cornel MC. Implementation of Pharmacogenetics in Primary Care: A Multi-Stakeholder Perspective. *Front Genet* 2020; **11**. doi:10.3389/fgene.2020.00010.

5 Dorfman EH, Brown Trinidad S, Morales CT, Howlett K, Burke W, Woodahl EL. Pharmacogenomics in diverse practice settings: implementation beyond major metropolitan areas. *Pharmacogenomics* 2015; **16**: 227–237.

6 Rafi I, Crinson I, Dawes M, Rafi D, Pirmohamed M, Walter FM. The implementation of pharmacogenomics into UK general practice: a qualitative study exploring barriers, challenges and opportunities. *J Community Genet* 2020; **11**: 269–277.

7 Asiedu GB, Finney Rutten LJ, Agunwamba A, Bielinski SJ, st. Sauver JL, Olson JE *et al.* An assessment of patient perspectives on pharmacogenomics educational materials. *Pharmacogenomics* 2020; **21**: 347–358.

8 Bright D, Worley M, Porter BL. Patient perceptions of pharmacogenomic testing in the community pharmacy setting. *Research in Social and Administrative Pharmacy* 2021; **17**: 744–749.

9 Sweet K, Hovick S, Sturm AC, Schmidlen T, Gordon E, Bernhardt B *et al.* Counselees’ Perspectives of Genomic Counseling Following Online Receipt of Multiple Actionable Complex Disease and Pharmacogenomic Results: a Qualitative Research Study. *J Genet Couns* 2017; **26**: 738–751.

10 Waldman L, Shuman C, Cohn I, Kaiser A, Chitayat D, Wasim S *et al.* Perplexed by PGx? Exploring the impact of pharmacogenomic results on medical management, disclosures and patient behavior. *Pharmacogenomics* 2019; **20**: 319–329.

11 Haddy CA, Ward HM, Angley MT, McKinnon RA. Consumers’ views of pharmacogenetics—A qualitative study. *Research in Social and Administrative Pharmacy* 2010; **6**: 221–231.

Current Genes & Health Research Team (in alphabetical order by surname):   Shaheen Akhtar, Mohammad Anwar, Elena Arciero, Omar Asgar, Samina Ashraf, Saeed Bidi, Gerome Breen, Raymond Chung, David Collier, Charles J Curtis, Shabana Chaudhary, Megan Clinch, Grainne Colligan, Panos Deloukas, Ceri Durham, Faiza Durrani, Fabiola Eto, Sarah Finer, Joseph Gafton, Ana Angel Garcia, Chris Griffiths, Joanne Harvey, Teng Heng, Sam Hodgson, Qin Qin Huang, Matt Hurles, Karen A Hunt, Shapna Hussain, Kamrul Islam, Vivek Iyer, Ben Jacobs, Ahsan Khan, Cath Lavery, Sang Hyuck Lee, Robin Lerner, Daniel MacArthur, Daniel Malawsky, Hilary Martin, Dan Mason, Rohini Mathur, Mohammed Bodrul Mazid, John McDermott, Caroline Morton, Bill Newman, Elizabeth Owor, Asma Qureshi, Samiha Rahman, Shwetha Ramachandrappa, Mehru Reza, Jessry Russell, Nishat Safa, Miriam Samuel, Michael Simpson, John Solly, Marie Spreckley. Daniel Stow, Michael Taylor, Richard C Trembath, Karen Tricker, Nasir Uddin, David A van Heel, Klaudia Walter, Caroline Winckley, Suzanne Wood, John Wright, Julia Zollner.
